# Phytochemical analysis and insight into insecticidal and antifungal activities of Indian hawthorn leaf extract

**DOI:** 10.1038/s41598-023-43749-9

**Published:** 2023-10-11

**Authors:** Wael M. Khamis, Said I. Behiry, Samy A. Marey, Abdulaziz A. Al-Askar, Ghoname Amer, Ahmed A. Heflish, Yiming Su, Ahmed Abdelkhalek, Mohamed K. Gaber

**Affiliations:** 1 Plant Protection Research Institute, Agriculture Research Center, Al-Sabhia, Alexandria, 21616 Egypt; 2 Agricultural Botany Department, Faculty of Agriculture (Saba Basha), Alexandria University, Alexandria, 21531 Egypt; 3 King Saud University, P.O. Box 2455, 11451 Riyadh, Saudi Arabia; 4 Department of Botany and Microbiology, College of Science, King Saud University, P.O. Box 2455, 11451 Riyadh, Saudi Arabia; 5 Plant Pathology Department, Faculty of Agriculture, Damanhour University, Damanhour, 22516 Egypt; 6 Utah Water Research Laboratory, Department of Civil and Environmental Engineering, Utah State University, Logan, UT 84341 USA; 7 Plant Protection and Biomolecular Diagnosis Department, ALCRI, City of Scientific Research and Technological Applications, New Borg El Arab City, 21934 Alexandria Egypt; 8 Plant Production Department, Faculty of Agriculture (Saba Basha), Alexandria University, Alexandria, 21531 Egypt

**Keywords:** Biochemistry, Biotechnology, Plant sciences

## Abstract

Fungicides or insecticides are popular means of controlling a variety of pathogens and insect pests; however, they can cause harmful effects on both human health and the environment. Different researchers have suggested using plant extracts, which have shown promise in managing fungi and insects. The purpose of this investigation was to explore the antifungal activities of an acetone extract made from the leaves of Indian Hawthorn (HAL) against phytopathogens that are known to harm maize crops, *Fusarium verticillioides* (OQ820154) and *Rhizoctonia solani* (OQ820155), and to evaluate the insecticidal property against *Aphis gossypii* Glover aphid. The HAL extract demonstrated significant antifungal activity against the two fungal pathogens tested, especially at the high dose of 2000 µg/mL. Laboratory tests on the LC_20_ of HAL extract (61.08 mg/L) versus buprofezin 25% WP (0.0051 mg/L) were achieved on *A. gossypii* Glover. HAL extract diminished the nymph's production over 72 h and their total reproductive rate. This extract was like buprofezin 25% WP in decreasing the daily reproductive rate, reproductive period, and mean survival percentage. Nevertheless, the newly-born nymphs of treated females with HAL extract attained the highest reduction in survival percentage at 46.00%. Equalized prolongations on the longevity of nymphs to 9.33, 8.33, and 7 days and the total life cycle to 15.00, 14.00, and 12.67 days were realized by HAL extract, buprofezin 25% WP, and the control, respectively. The olfactory choice test on the aphids showed the minimum attraction rate to HAL extract. The HPLC of HAL extract comprised an abundance of phenolic compounds (ferulic acid, gallic acid, 4-hydroxybenzoic acid, salicylic acid, ellagic acid, and pyrogallol), and the concentrations of these compounds vary widely, with salicylic acid being the most concentrated at 25.14 mg/mL. Among the flavonoids, epicatechin has the highest concentration at 11.69 mg/mL. The HAL extract GC–MS consists of various organic compounds, including sesquiterpenes, cyclopropenes, fatty acids, steroids, alcohols, ketones, esters, bufadienolides, opioids, and other organic compounds. The most abundant compounds in the sample are *n*-hexadecanoic acid (12.17%), followed by 5α, 7αH, 10α-eudesm-11-en-1α-ol (9.43%), and cis-13-octadecenoic acid (5.87%). Based on the findings, it can be inferred that the HAL extract may be a viable option for plants to combat both fungal and insect infestations. This presents an encouraging prospect for utilizing a natural and sustainable approach toward long-term pest management in plants.

## Introduction

Indian hawthorn, or India hawthorn (*Rhaphiolepis indica* L.), is a plant species in the family Rosaceae. It is widely distributed throughout tropical and subtropical regions of Asia, including India, China, Japan, and Malaysia. The leaves, stems, and roots of Indian hawthorn have been shown to exhibit a range of pharmacological activities, including anti-inflammatory, antioxidant, hypoglycemic, anticancer, antimicrobial, and hypolipidemic effects, and are a therapeutic agent for treating various diseases^1,2^.

Regarding the maize crop, according to recent data, Egypt's maize production reached 7.4 million metric tons in 2022, indicating an increase from the 6.4 million metric tons produced in 2020. Throughout the period analyzed, the highest corn production levels were recorded in 2021 and 2022, while the lowest was reported in 2011 at 5.5 million metric tons. Worldwide, the maize crop is devastated by several pathogens, such as fungi of the *Fusaria* group, which are commonly associated with maize plants and cause diseases in various parts, including seedlings, roots, stalks, and kernels. *Fusarium verticillioides*, the most prevalent species infecting maize ears in Egypt, leads to ear or kernel rot disease, which can reduce yield and quality and result in mycotoxin accumulation in grain^3–5^. Also, the *Rhizoctonia solani* fungus is known to infect several other crops besides maize, causing various diseases such as root rot^6^.

Also, maize crops encounter different pests, including the cotton aphid, *Aphis gossypii* Glover (Hemiptera: Aphididae), which is the biggest destroyer of enormous economic crops^7–9^. Its direct sap feeding on the cell contents leads to yield loss. Moreover, it blocks the photosynthesis process caused by the extended superficial growth of black sooty mold resulting from the honeydew exertion of the cotton aphid^10^.

In recent years, medicinal plants and other natural products used to treat various pathogenic diseases have increased in popularity because of their potential to circumvent pesticide resistance and the unpleasant side effects associated with chemicals^11–13^. In light of these concerns, the search for natural, bio-based alternatives has gained momentum. Botanical extracts have emerged as potential candidates for their cost-effectiveness and inherent bioactive compounds that exhibit antifungal and insect-repellent properties. Studies about the botanical compounds responsible for the biological features of herbal plants have been inspired by the discovery of chemical compounds produced by these plants^13–16^. Indian hawthorn is one of these plants according to its phytochemical profile, which contains a variety of bioactive compounds, including flavonoids, triterpenoids, and phenolic acids. The most abundant flavonoids identified in the plant are quercetin, kaempferol, and their glycosides. Triterpenoids such as ursolic and oleanolic acids have also been isolated from the plant^1^.

There have been several research studies conducted to investigate the efficacy of various extracts against *F. verticillioides* and *R. solani*, and the results have been promising, indicating that these extracts possess antifungal activities^17–20^. The extracts from different parts of Indian hawthorn species contain unique components compared to their counterparts in other plants that possess distinctive efficiency against many insects, which may qualify them to have an exterminatory role in insecticidal activity^1^. To compare the extract results, a buprofezin compound was widely used against piercing-sucking hemipteran pests. Buprofezin possesses a particular effect on the biological aspects represented in the diminishing of the chitin contents in successive generations of cotton aphids^21,22^.

The objectives of this research were to isolate and identify the specific pathogens responsible for causing maize ear rot and root rot, assess the insecticidal and antifungal effects of an acetone extract obtained from Indian hawthorn leaves (HAL) against *Aphis gossypii* Glover and the isolated fungal pathogens, and identify the primary constituents of the extract using HPLC and GC–MS analysis.

## Materials and methods

### Fungal strains used in the study

In this study, the fungal strains utilized were obtained from maize and identified morphologically at the genus level using a fungal identification manual. To achieve molecular identification, the ITS region was amplified utilizing universal primers ITS1 and ITS4 with previously reported PCR conditions. Following amplification, the PCR products underwent purification and sequencing through Sanger technology. The resultant sequences were annotated and compared to the GenBank database via alignment with NCBI, and the fungal sequences were subsequently submitted to the GenBank database to obtain accession numbers^23–25^.

### HAL extract setting up

Fresh Indian hawthorn (HAL) leaves were collected from the Faculty of Agriculture (Saba Basha) Garden, Alexandria, Egypt, and cleaned thoroughly to ensure that they had not been exposed to any harmful chemicals. The plant material was entirely dried by spreading it on a tray and leaving it in a well-ventilated area. Once the plant material was fully dry, it was ground into a fine powder using a grinder.

The powdered HAL (90 g) was then transferred into a clean glass jar, and acetone (900 mL, 99% ACS reagent purity) was poured into the jar. The jar was sealed tightly and shaken vigorously to ensure that the acetone was thoroughly mixed with the plant material. The mixture was then left to soak for 24 h at room temperature while being shaken occasionally. This allowed the acetone to extract the desired compounds from the plant material. After 24 h, the mixture was filtered through filter paper to remove any solid particles. This step was repeated until the extract was free of any visible particles. The filtered extract was then transferred into a shallow glass dish and allowed to evaporate completely at room temperature in a fume hood with a rotary evaporator at 40 °C (DAIHAN-RVE-05, Singapore). The residue left behind was scraped into a clean, dry container. The HAL extract was stored in a cool, dry place in a dark-colored glass container to prevent light from breaking down the active compounds^26^. The yield of the extract was calculated as: Yield extract = [HAL dry extract after solvent removal (g)/dry HAL powdered material used in extraction (g)] × 100^27^.

### HAL effect on fungal hyphal growth using food poison technique

To investigate the effect of HAL extract on fungal hyphal growth, a food poisoning technique was employed by adding the extract directly to the culture medium. First, a culture of the test fungus was grown on Potato Dextrose Agar (PDA) plates until it formed a uniform mat of mycelium. The mycelium was then cut into small pieces using a sterile scalpel and transferred onto fresh PDA plates containing different concentrations of HAL extract (500, 1000, and 2000 μg/mL). As a control, PDA plates with no HAL extract were prepared. All plates were incubated at the optimal temperature for the test fungus for 7 days. After this period, the diameter of the fungal colony was measured using a ruler. The hyphal growth (mm) was also measured to determine the effect of the extract on the development of the fungus. This process was repeated three times for each concentration of the extract^28,29^.

### HAL extract insecticidal activity

#### Tested compounds

- Acetone extract of Indian hawthorn leaves
- Buprofezin (Solano 25% WP belongs to the benzoylphenyl urease group, a chitin synthesis inhibitor) was obtained from El-Helb Pesticides and Chemical Co.

#### Rearing units of cotton aphid

Individuals of *Aphis gossypii* Glover were collected from the available host plants. The collected aphids were allowed to grow in a rearing unit comprised of cotton seedlings (variety Giza 86) in polypropylene pots (diameter, 10 cm; height, 8 cm), covered up with a muslin jacket. Each rearing unit was placed into a growth incubator, digitally outfitted with a thermostat and a hygrometer, to maintain stable conditions at 25 ± 2 °C, 60% RH, and 16 h of lightning, according to Gaimari and Turner^30^. Newly ripe seedlings must be replaced by malformed ones resulting from the feeding of aphids. More seedlings must be added to meet the exacerbation of aphid growth. A laboratory strain (LS) of cotton aphids was attained after approximating seven generations^31^.

#### Toxicity assay

The toxicity of acetone extract of Indian hawthorn and buprofezin 25% WP against *A. gossypii* Glover was carried out by using the slide-dipping method invented by Stirbly et al.^32^. Each tested compound had six gradual concentrations in distilled water. A double-faced piece of Scotch tape was stacked on one surface of a glass slide. A delicate brush was used to load 20 adult females over the surface of the Scotch tape. For each concentration in the tested compound, the slide was dipped for 10 s. Meanwhile, the control slide was dipped in distilled water. Three replicated slides were used for each concentration. The treated slides were incubated under the same laboratory conditions. Every 24 h, the numbers of dead and living aphids were recorded using a stereoscopic microscope. Mortality percentages were corrected according to the formula of Abbott^33^. The results were subjected to probit analysis^34^.

#### Biological aspects

A susceptibility test using the technique of leaf disks on cotton seedlings was accomplished according to Insecticide Resistance Action Committee (IRAC)^35^. The leaf disks were dipped in the sub-lethal concentration (LC_20_) of each tested compound and distilled water for control. One leaf disk was attached to the whole cohesive surface of an agar gel 1% filled the bottom of a cup (diameter, 7 cm; height, 2.5 cm).

##### Adult female

Twenty uniformed adult female cotton aphids (LS) were loaded gently onto the leaf disk. Each cup was capped with micro-hole gauze and tied with a rubber band. Each treatment was replicated with triple cups. The treated cups were incubated under the foregoing laboratory conditions. The affected aphids were monitored every 24 h using a stereoscopic microscope. The daily production of nymphs, total reproduction, survival percentage, and longevity of adult females were recorded after exposure to LC_20_ of each treatment according to Michelotto et al.^36^.

##### The first-born nymphs from treated females

Twenty of the first-born nymphs (produced from the treated females with each tested compound at LC_20_ in comparison to the control) were monitored for their daily and total survival rate and longevity as performed by Michelotto et al*.*^36^.

#### Choice test of olfactory response

Adult cotton aphids were submitted to an olfactometer set designed by Jaba et al.^37^. Twenty pre-starved adults were placed in a central cell connected to each of three lateral tubes through a short tube. Each lateral tube contained 3 g of fresh, tender leaf. These leaves were dipped in LC_20_ of the tested compounds for 10 s versus the control leaf in distilled water. Air was allowed to pass over the hosted leaves in each lateral tube using an air compressor through slime hoses attached. The three-hosted leaves were inset at the same time in the lateral tube. Each treatment had three replicates of olfactometer sets. The number of aphids that responded to each tube was recorded every hour for up to 4h. The retention time of aphids needed to accomplish their movement toward each tube was quantified at the end of the 4th h. The equation of attraction response percentage was expressed by Weeks et al.^38^ as follows:

$$\mathrm{Total attraction percentage }= \frac{\left[100\times \left(T+C\right)\right]}{N}$$

where T is the number of attracted aphids to the host tube treated by tested compounds; C is the number of attracted aphids to the control tube; and N is the total number of aphids submitted to the olfactometer set.

### High-performance liquid chromatography analysis

High-performance liquid chromatography (HPLC) is a widely used analytical technique for the separation, identification, and quantification of individual components in a mixture. In this study, HPLC Agilent 1260 was used to analyze the target compounds in the samples. Sample preparation involved extracting the target compounds from the sample matrix and filtering it to remove any impurities. The column selected was possessed dimensions of 4.6 mm in diameter and 250 mm in length, featuring a particle size of 5 μm. The mobile phase used consisted of two key components: H_2_O (A) and 0.05% CF_3_COOH in CH_3_CN (B), delivered at a constant flow rate of 0.9 mL/min.

The HPLC system was calibrated using standard compounds of known concentration and purity before running the samples. The samples were then injected into the HPLC system with an injection volume of 5 μL, and a column temperature meticulously maintained at 40 °C. To facilitate the effective separation of sample constituents, we implemented a gradient elution program with distinct phases. Precisely, during the first 5 min, the mobile phase comprised 80% solvent A and 20% solvent B; from 5 to 8 min, it transitioned to 60% solvent A and 40% solvent B; maintaining this composition from 8 to 12 min. Subsequently, from 12 to 16 min, the mobile phase returned to its initial state with 82% solvent A and 18% solvent B, and finally, from 16 to 20 min, it remained consistent with 82% solvent A and 18% solvent B.. The target compounds were separated based on their physicochemical properties and interacted with the stationary phase of the column. The separated compounds were detected by a UV–visible detector, specifically monitored at 280 nm, which generated a signal proportional to the concentration of the compound. The output from the detector was recorded and analyzed using specialized software. The chromatogram showed the retention time and peak area of each compound, which were used to identify and quantify the components in the sample. The HPLC analysis results showed that the samples contained the target compounds at the expected concentrations. The precision and accuracy of the HPLC analysis were validated by the low standard deviation and high recovery rates of the standard compounds. These results demonstrate the suitability of HPLC analysis for the characterization of complex mixtures and the determination of the purity of a compound^39^.

### Gas chromatography–mass spectrometry analysis

Gas chromatography-mass spectrometry (GC–MS) 7000D instrument, manufactured by Agilent Technologies in Santa Clara, CA, USA, was used to analyze the target organic compounds in the sample. Before analysis, the sample was extracted and purified to remove any impurities. The purified sample was injected into the GC column packed with a 5% diphenyl/95% dimethylpolysiloxane stationary phase (HP-5MS), which was coated with a stationary phase that separated the target compounds based on their physical properties. The GC column was heated to separate the target compounds in the sample mixture, and the separated compounds were carried by the carrier gas, helium (purity, 99.99%), flowing at a constant rate of 1 mL/min., through the column and into the mass spectrometer. In the mass spectrometer, the separated compounds were ionized by electron impact ionization,which was set at 70 electronvolts (eV), and the scan time was fixed at 0.2 s, producing charged fragments that were separated based on their mass-to-charge ratio (*m/z*) range from 40 to 600. The mass spectrometer detected the charged fragments and generated a mass spectrum, which was a unique fingerprint of the compounds in the sample. For each sample injection, a volume of 1 μL was introduced, employing a split ratio of 10:1. The injector temperature was held steady at 250 °C throughout the analysis. The temperature program of the column oven began at 50 °C for an initial 3-min period, followed by a linear ramp of 10 °C per minute until reaching 280 °C. Subsequently, the temperature was raised to 300 °C and maintained for an additional 10 min.

The mass spectra were analyzed using specialized software, which identified the compounds based on their fragmentation patterns and retention times. The output from the mass spectrometer was recorded and analyzed by different liberires, including Wiley Registry 8E and Replib, and the relative abundance and mass-to-charge ratio of each fragment were used to identify and quantify the components in the sample.

### Statistical analyses

Statistical analyses were performed using SAS software. An ANOVA (Analysis of Variance) was performed to assess overall treatment effects. A significance level of 0.05 was used, and the LSD (Least Significant Difference) test was employed to determine significant differences among treatment groups^40^.

### Statement of permissions and/or licenses for collection of plant or seed specimens

The authors declare that the maize specimens used in this study are publicly accessible maize cultivars, and we were assigned to NCBI respiratory under accession numbers OQ820154 and OQ820155.

### Plant guidelines

Experimental research and field studies on plants, including the collection of plant material, comply with relevant institutional, national, and international guidelines and legislation—Formal ethical approval is not required.

## Results

### The procedure for the isolation and characterization of fungal strains

After investigating the maize plant's ear and root, two types of pathogens were isolated and identified as *Fusarium* and *Rhizoctonia* species. To further identify and classify these fungi, molecular analysis was performed by sequencing the amplified ITS region, which showed 100% homology with *Fusarium verticillioides* and *Rhizoctonia solani*. To make these results widely available, the sequences for the two fungal organisms were deposited in the NCBI database and assigned accession numbers OQ820154 for *F. verticillioides* and OQ820155 for *R. solani*.

### The impact of HAL extract on fungal strains

The data presented in Table 1 show the effect of Indian hawthorn leaf acetone extract at different concentrations on the hyphal growth of two fungal pathogens, *R. solani,* and *F. verticillioides*. The results are shown in terms of mean hyphal growth in millimeters (mm). Based on the results, the HAL extract had a significant inhibitory effect on the hyphal growth of both fungal pathogens, with increasing concentrations leading to greater inhibition. The mean hyphal growth for both pathogens decreased significantly as the concentration of the extract increased from 500 to 1000 μg/mL and 2000 μg/mL, respectively.

**Table 1:** The fungal growth of *Fusarium verticillioides* and *Rhizoctonia solani* under in vitro conditions treated with Indian hawthorn leaf (HAL) extract.

Indian hawthorn extract Conc. (μg/mL)	Hyphal growth (mm)	
	*Rhizoctonia solani*	*Fusarium verticillioides*
500	57.67 b ± 0.24	57.33 b ± 0.85
1000	32.67 c ± 1.25	45.00 c ± 1.41
2000	15.33 d ± 0.47	12.33 d ± 0.62
Negative control	90.00 a ± 0.00	90.00 a ± 0.00

If the letters in each column are different, it indicates that there is a significant difference between the data sets within that column, as determined by the LSD test with a probability level of 0.05.

For *R. solani*, the mean hyphal growth decreased from 57.67 to 32.67 mm and 15.33 mm at 500 μg/mL, 1000 μg/mL, and 2000 μg/mL, respectively. Similarly, for *Fusarium verticillioides*, the mean hyphal growth decreased from 57.33 to 45.00 mm and 12.33 mm at 500 μg/mL, 1000 μg/mL, and 2000 μg/mL, respectively. The results of the statistical analysis revealed that the mean hyphal growth values were significantly different among the concentrations for both fungal pathogens. The mean hyphal growth values for the highest concentration (2000 μg/mL) were significantly different from those of the other concentrations. Furthermore, the mean hyphal growth values for the negative control (Nc) were significantly different from those of all other treatments, indicating that the extract had a significant inhibitory effect on the fungal pathogens. In summary, the HAL extract demonstrated significant antifungal activity against the two fungal pathogens tested. Further studies may be necessary to determine the specific antifungal compounds present in the extract and to evaluate its potential as a natural alternative to synthetic fungicides in plant disease management (Table 1).

### Toxicity of the tested HAL extract on the adults of *Aphis gossypii*

Toxic effect results under laboratory conditions were achieved to determine the LC_20_ and LC_50_ values of HAL extract compared to buprofezin 25% WP on the adult stage of *A. gossypii* Glover after 24 h of exposure (Table 2). Buprofezin 25% WP exhibited toxicity values of 0.022 and 0.0051 mg/L higher than the toxicity of HAL extract at 245.35 and 61.08 mg/L at LC_50_ and LC_20_, respectively.

**Table 2:** Sub-lethal concentrations of the tested compounds on the adults of *Aphis gossypii* Glover along 24 h of exposure.

Tested compound	Lethal concentration (mg/L)		Confidence margins (mg/L)	Slope ± SE**	χ^2^***	df	N****
Indian hawthorn acetone extract	LC_20_	61.08	(41.57–89.75)	0.41 ± 0.06	0.31	4	360
	LC_50_	245.35	(194.95–308.78)				
Buprofezin 25% WP	LC_20_	0.0051	(0.0033–0.0077)	0.11 ± 0.04	5.95	4	360
	LC_50_	0.022	(0.017–0.028)				

**Standard error, ***Chi square, ****Total insect individuals assigned for the toxicity assay.

### Biological aspects of adults of *Aphis gossypii* and their newly born nymphs

#### Adult female

Data on the daily production of the born nymphs per female, reproductive rate, survival percentage, and longevity were evaluated after the exposure of *A. gossypii* adult female to LC_20_ of the tested compounds (Table 3).

**Table 3:** Biological effects of the tested compounds at LC_20_ on the reproduction rate and the longevity of the treated adult female of *Aphis gossypii.*

Treatments	Daily production of born nymphs/female (every 24 h after exposure) ± SD^a^				Reproductive rate (days) ± SD		Reproductive period (day)	The mean of survival %	Longevity (days)
	24	48	72	96	Daily	Total			
Indian hawthorn extract	0.73c ± 0.08	0.62b ± 0.04	0.47b ± 0.41	0.27a ± 0.46	0.52b ± 0.01	2.08c ± 0.02	2.67b ± 0.58	39.29b ± 3.57	5.67a ± 0.58
Buprofezin 25% WP	1.38b ± 0.13	0.50b ± 0.18	0.36b ± 0.22	0.00a ± 0.00	0.56b ± 0.05	2.24b ± 0.59	3.00ba ± 0.00	41.25b ± 5.45	6.33a ± 0.58
Control	2.07a ± 0.23	4.19a ± 0.38	5.84a ± 0.84	3.67a ± 3.26	3.94a ± 1.55	15.77a ± 2.00	3.67a ± 0.58	76.33a ± 5.39	5.67a ± 0.58

^a^*SD* Standard deviation. The means of data in each column linked with the same alphabetical characters are not significantly different based on the LSD_0.05_.

Indian hawthorn extract had a potent effect on the born nymphs production per female (0.73) compared to buprofezin 25% WP (1.38) and the control (2.07) at 24 h after exposure. Meantime, the results of nymph production per female in HAL extract were comparable to buprofezin 25% WP compared to the control at 48 and 72 h after exposure. All the treatments had no significant differences in the nymph production at 96 h after exposure. The total reproductive rate of HAL extract was significantly decreased (2.08) more than buprofezin 25% WP (2.24) when compared to its counterpart rate in control (15.77). There were no significant differences in the longevity of the adult aphids exposed to all treatments. Significant decreases for HAL extract were comparable to buprofezin 25% WP on the daily reproductive rate, reproductive period, and mean survival percentages compared to the control.

#### First-day-born nymphs from treated adults

Daily and mean survival percentages and longevity data were accomplished on the first day-born nymphs 7 days after exposure to LC_20_ of the tested compounds (Table 4).

**Table 4:** Biological effects of the tested compounds at LC_20_ on the longevity and survival percentages of the newly born nymphs of *Aphis gossypii.*

Treatments	Daily survival% (every 24 h after exposure) ± SD^a^							Mean of survival %	Longevity (days)
	24	48	72	96	120	144	168		
Indian hawthorn extract	90.00b ± 5.00	78.33b ± 2.89	33.33c ± 2.89	20.00b ± 5.00	8.33b ± 7.64	0.00b ± 0.00	0.00b ± 0.00	46.00c ± 3.61	9.33a ± 1.15
Buprofezin 25% WP	100.00a ± 0.00	88.33ba ± 12.58	43.33b ± 2.89	15.00b ± 5.00	0.00b ± 0.00	0.00b ± 0.00	0.00b ± 0.00	61.67b ± 3.61	8.33ba ± 0.58
Control	100.00a ± 0.00	100.00a ± 0.00	100.00a ± 0.00	100.00a ± 0.00	100.00a ± 0.00	98.33a ± 2.89	91.67a ± 7.64	98.57a ± 1.43	7.00b ± 0.00

^a^*SD* Standard deviation. If the letters in each column are different, it indicates that there is a significant difference between the data sets within that column, as determined by the LSD test with a probability level of 0.05.

The daily survival nymphs in HAL extract were vigorously affected with percentages of 90.00, 78.33, and 33.33% compared to buprofezin 25% WP at 100.00, 88.33, and 43.33%, and the control that fulfilled the highest survival percentages at 100.00% along 24, 48, and 72 h after exposure, respectively. Equipollent reductions occurred in the daily survival percentages of nymphs in both treatments of HAL extract and buprofezin 25% WP, which were significantly less than the control at 96, 120, 144, and 168 h of exposure. HAL extract had a significant reduction in the mean survival percentages of nymphs (46.00%) that surpassed buprofezin 25% WP (61.67%) and the control (98.57%). The longevity of nymphs was prolonged in HAL extract to 9.33 days, followed by buprofezin 25% WP at 8.33 days, while in the control it was 7 days.

#### Total life of *Aphis gossypii*

The current results of the tested compounds at LC_20_ exhibited equipollent prolongations on the total life cycle for HAL extract at 15 days and buprofezin 25% WP at 14 days, compared to the highest life duration in control at 12.67 days (Table 5).

**Table 5:** Biological effects of the tested compounds at LC_20_ on the total life cycle of *Aphis gossypii.*

Treatments	Total life cycle ± SD
Indian hawthorn extract	15.00 a ± 1.00
Buprofezin 25% WP	14.00 ab ± 0.58
Control	12.67 b ± 0.58

*SD* Standard deviation. If the letters in each column are different, it indicates that there is a significant difference between the data sets within that column, as determined by the LSD test with a probability level of 0.05.

### Olfactory response of the choice test on the adults of Aphis gossypii

**Figure 1:**
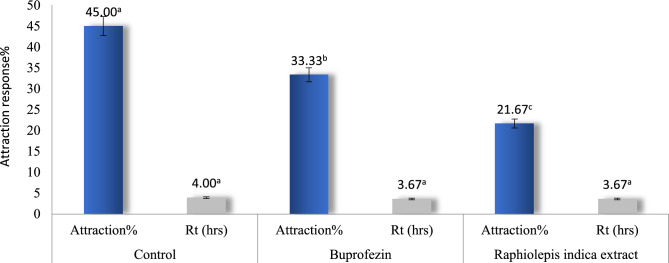
Olfactory choice test of the tested compounds at LC_20_ on the attraction response of adults *Aphis gossypii* at the 4th h of exposure.

The lateral chamber that hosted the treated leaves with *R. indica* extract possessed the lowest attraction percentage on aphid individuals (21.67%) compared to buprofezin 25% WP (33.33%) and control treatment (45.00%). There were no significant differences between the treatments for the retention times (Rt) of aphid individuals needed to accomplish their orientations at 4 h of test duration (Fig. 1).

### HAL extract yield and polyphenolic content

**Figure 2:**
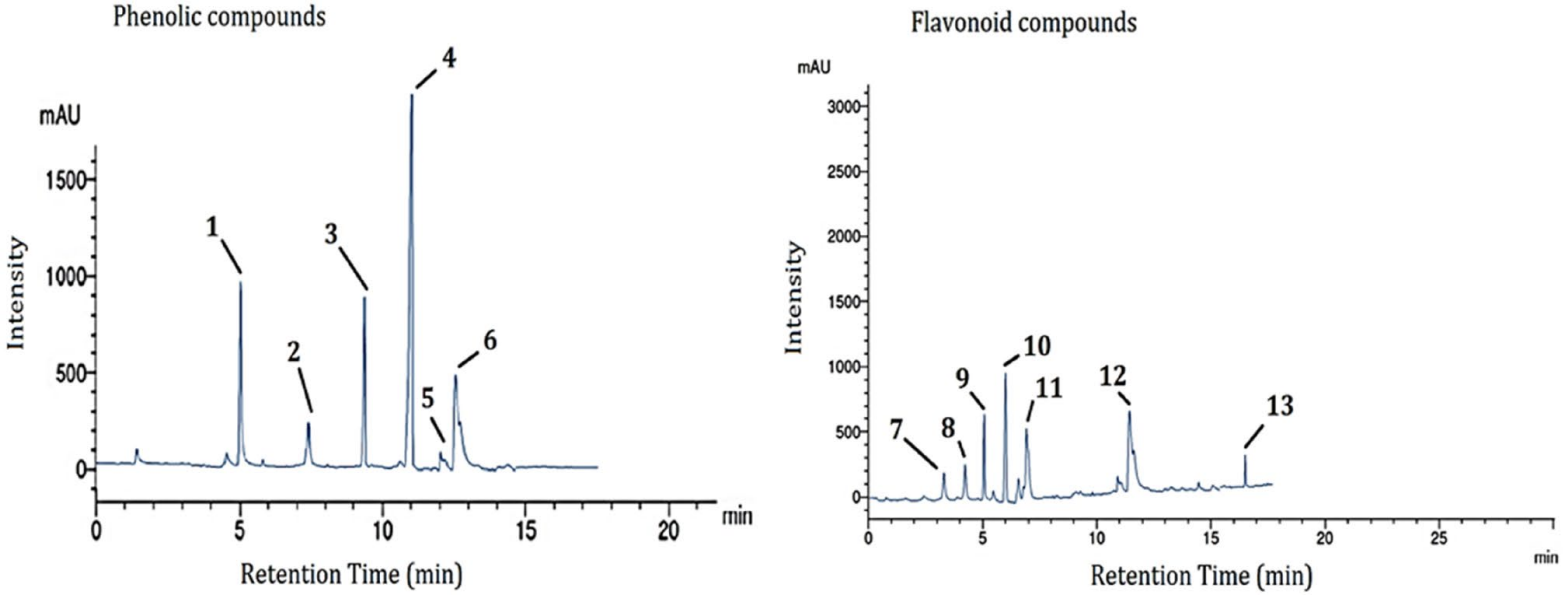
The HPLC chromatograms of Indian hawthorn extract show the peaks of the following phenolic compounds identified at a wavelength of 284 nm: 1 = ferulic acid, 2 = gallic acid, 3 = 4-hydroxybenzoic acid, 4 = salicylic acid, 5 = ellagic acid and 6 = pyrogallol; at a wavelength of 273 nm, the peaks represent the following flavonoids: 7 = apigenin, 8 = rutin, 9 = chrysin, 10 = epicatechin, 11 = quercetin and 12 = 7-oh flavone and 13 = acacetin.

The yield of HAL extract obtained was found to be 4.33% for every 90 g of initial ground material used. The data in Table 6 and Fig. 2 represent the retention time (in minutes), compounds, and their respective concentrations (mg/mL) for various phenolics and flavonoids. The compounds listed under phenolics are ferulic acid, gallic acid, 4-hydroxybenzoic acid, salicylic acid, ellagic acid, and pyrogallol. The compounds listed under flavonoids are apigenin, rutin, chrysin, epicatechin, quercetin, 7-OH flavone, and acacetin.

**Table 6:** Phenolic and flavonoid components detected in Indian hawthorn extract.

Retention time (min)	Compounds	Concentration (mg/mL)
Phenolics		
5.0	Ferulic acid	12.04
7.3	Gallic acid	4.25
9.0	4-Hydroxybenzoic acid	10.33
11.0	Salicylic acid	25.14
12.0	Ellagic acid	0.55
13	Pyrogallol	6.18
Flavonoids		
3.0	Apigenin	2.33
4.0	Rutin	3.42
5.0	Chrysin	8.14
6.0	Epicatechin	11.69
7.0	Quercetin	7.18
11.88	7-OH flavone	9.15
16.8	Acacetin	4.66

The concentrations of these compounds vary widely, with salicylic acid being the most concentrated at 25.14 mg/mL, while ellagic acid has the lowest concentration at 0.55 mg/mL. Among the flavonoids, epicatechin has the highest concentration at 11.69 mg/mL, while apigenin has the lowest concentration at 2.33 mg/mL. Other compounds present in lower concentrations include gallic acid (4.25 mg/mL), pyrogallol (6.18 mg/mL), and chrysin (8.14 mg/mL). The extract also contains various flavonoids, including apigenin (2.33 mg/mL), rutin (3.42 mg/mL), quercetin (7.18 mg/mL), 7-OH flavone (9.15 mg/mL), and acacetin (4.66 mg/mL).

### GC–MS content of Indian hawthorn (HAL) extract

**Figure 3:**
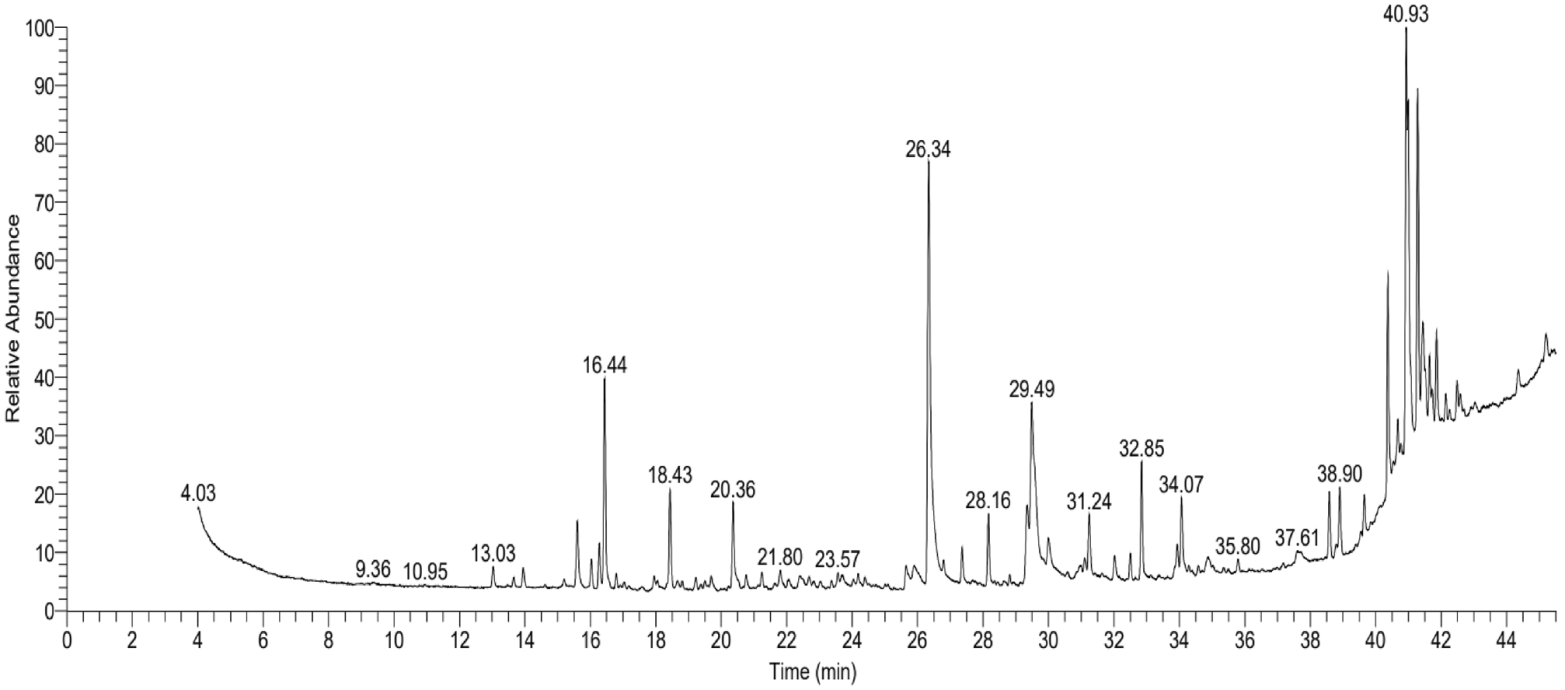
Identified phytocompounds in the acetone leaf extract of Indian hawthorn using gas chromatography-mass spectrometry.

From Table 7 and Fig. 3, we concluded that the HAL extract GC–MS consists of various organic compounds, including sesquiterpenes, cyclopropenes, fatty acids, steroids, alcohols, ketones, esters, bufadienolides, opioids, and other organic compounds. The most abundant compounds in the sample are n-hexadecanoic acid (12.17%), followed by 5α,7αH,10α-eudesm-11-en-1α-ol (9.43%), and cis-13-octadecenoic acid (5.87%).

**Table 7:** GC–MS results of Indian hawthorn acetone leaf extract*.*

RT (min)	RA%	Compound	Class
13.03	0.59	α-copaene	Sesquiterpene
13.95	0.58	(3S,3aR,3bR,4S,7R,7aR)-4-Isopropy l-3,7-dimethyloctahydro-1H-cyclopen ta[1,3]cyclopropa[1,2]benzen-3-ol	Terpene
15.60	1.90	Aromandendrene	Sesquiterpene
16.03	0.78	1H-Cyclopenta[1,3]cyclopropa[1,2]b enzene, octahydro-7-methyl-3-methylene-4-(1 -methylethyl)-, [3aS-(3aà,3bá,4á,7à,7aS*)]-	Cyclopropene
16.27	1.15	Longifolene-(V4)	Sesquiterpene
16.44	5.36	1H-Cycloprop[e]azulene, 1a,2,3,5,6,7,7a,7b-octahydro-1,1,4,7-tetramethyl-, [1aR-(1aà,7à,7aá,7bà)]-	Cyclopropene
16.79	0.36	(3S,3aR,3bR,4S,7R,7aR)-4-Isopropy l-3,7-dimethyloctahydro-1H-cyclopen ta[1,3]cyclopropa[1,2]benzen-3-ol	Terpene
18.43	2.80	(−)-Globulol	Sesquiterpene
20.36	2.44	Caryophyllene oxide	Sesquiterpene
21.24	0.49	2-(4-Nitrobutyryl)cyclooctanone	Ketone
21.80	0.58	Androstan-17-one, 3-ethyl-3-hydroxy-, (5α)-	Steroid
23.57	0.42	4,7-Octadecadiynoic acid, methyl ester	Fatty acid
25.64	0.78	Cyclopropanebutanoic acid, 2-[[2-[[2-[(2-pentylcyclopropyl)meth yl]cyclopropyl]methyl]cyclopropyl] methyl]-, methyl ester	Cyclopropane
26.34	12.17	*n*-Hexadecanoic acid	Fatty acid
27.35	1.05	13-Heptadecyn-1-ol	Alcohol
28.16	1.88	Palmitic Acid, TMS derivative	Fatty acid
29.33	1.63	9,12-Octadecadienoic acid (Z,Z)-	Fatty acid
29.49	5.87	*cis*-13-Octadecenoic acid	Fatty acid
30.00	0.98	Octadecanoic acid	Fatty acid
31.10	0.45	11-*cis*-octadecenoic acid 1tms	Fatty acid
31.24	1.68	Glycerol 1-palmitate	Glycerol ester
32.02	0.70	Glycidyl oleate	Ester
32.50	0.73	Resibufogenin	Bufadienolide
32.85	3.16	4a,7a-Epoxy-5H-cyclopenta[a]cyclop ropa[f]cycloundecen-4(1H)-one, 1a,6,7,10,11,11a-hexahydro-7,10,11- trihydroxy-1,1,3,6,9-pentamethyl	Terpene
33.93	0.79	α-N-Normethadol	Opioid
34.07	2.09	Oleic anhydride	Fatty acid
38.59	1.87	1-Heptatriacotanol	Alcohol
38.90	2.01	Drostanolone	Steroid
39.65	1.09	Androstan-17-one, 3-ethyl-3-hydroxy-, (5α)-	Steroid
40.37	5.43	Bicyclo[4.3.0]nonane, 1-isopropenyl-4,5-dimethyl-5-phenyl sulfonylmethyl	Organic compound
40.68	1.14	2H-3,9a-Methano-1-benzoxepin, octahydro-2,2,5a,9-tetramethyl-, [3R-(3à,5aà,9à,9aà)]-	Organic compound
40.77	0.30	Loperamide	Opioid
40.93	9.43	5α,7αH,10α-Eudesm-11-en-1α-ol	Terpene
41.00	5.85	Naphthalene, decahydro-4a-methyl-1-me thylene-7-(1-methylethyli dene)-, (4ar-trans)-	Organic compound
41.07	0.25	Arabinitol, pentaacetate	Carbohydrate
41.29	8.25	1-Isopropenyl-4,5-dimethylbicyclo[4. 3.0]nonan-5-ylmethyl phenyl sulfoxide	Sulfoxide
41.45	2.91	1,4-Methanoazulen-9-ol, decahydro-1,5,5,8a-tetramethyl-, [1R-(1à,3aá,4à,8aá,9S*)]-	Azulenol
41.51	0.95	9,19-Cyclolanostan-3-ol, 24,24-epoxymethano-, acetate	Cyclolanostanol
41.64	1.47	Silane, trimethyl[[(3α)-stigmast-5- en-3-yl]oxy]-	Silane
41.72	0.80	9-Octadecenoic acid, 1,2,3-propanetriyl ester, (E,E,E)-	Fatty acid ester
41.86	2.41	9,12-octadecadienoic acid (z,z)-, 2,3-bis[(trimethylsilyl)oxy]propyl ester	Fatty acid ester
42.14	0.65	9-Octadecenoic acid, 1,2,3-propanetriyl ester, (E,E,E)-	Fatty acid ester
42.49	1.02	Cyclopentanepentanoic acid, 2-(3-oxooctyl)-3,5-bis[(trimethylsilyl) oxy]-, methyl ester, [1R-(1à,2á,3à,5à)]-	Fatty acid ester
42.58	0.76	Dasycarpidan-1-methanol, acetate (ester)	Alcohol ester
44.35	1.07	9-Octadecenoic acid, 1,2,3-propanetriyl ester, (E,E,E)-	Fatty acid ester
45.20	0.90	Linoleic acid ethyl ester	Fatty acid ethyl ester

*RT* Retention time, *RA* Relative abundance.

Several compounds appear to be present in relatively low amounts (below 1% relative abundance), including the ketone 2-(4-Nitrobutyryl)cyclooctanone, the steroid Androstan-17-one, 3-ethyl-3-hydroxy-, (5α)-, the fatty acid 11-cis-octadecenoic acid 1tms, and the opioid α-N-Normethadol, among others. Sesquiterpenes and fatty acids are the most common compound classes in the sample, with each class containing 8 compounds. The HAL extract contains several compounds with potential pharmacological activity, such as loperamide, resibufogenin, and α-N-normethadol, which are opioids; and (−)-globulol, caryophyllene oxide, and á-copaene, which have reported anti-inflammatory and analgesic properties.

## Discussion

Medicinal plants, which are recognized for their reliability, strength, and environmentally sustainable impact, are being used in the field of environmental management to combat plant diseases and reduce the harmful effects of synthetic antimicrobials. In this study, the acetone extract of Indian hawthorn leaves (HAL) was evaluated for its bioactive substances using various conventional assays. The results revealed the presence of a diverse range of phytochemical components, including salicylic acid, epicatechin, ferulic acid, hydroxybenzoic acid, *n*-hexadecanoic acid, fatty acid esters, and terpenes, which are known to be biologically active and effective against plant pathogens. These findings are consistent with those of previous studies and suggest that the botanical compound substances in the plant extract play a critical role in its biological activity^31,41,42^. Also, other researchers^43^ investigated the relationship between the plant extract's chemical constituents and its therapeutic properties.

After conducting HPLC and GC–MS analyses of HAL extract, we identified several highly potent molecules, including salicylic acid, epicatechin, ferulic acid, and *n*-hexadecanoic acid. These compounds have been extensively studied for their antimicrobial activities^44–46^. Literature suggests that salicylic acid (SA), a derivative of benzoic acid, is a key component in the resistance of plants against diseases, especially during the process of systemic acquired resistance^47^. While some studies have suggested that salicylic acid (SA) does not directly exhibit antifungal activity^48^, other research has indicated that SA can affect various stages of fungal development. For example, SA has been found to hinder conidial germination and hyphae development in *Sphaerotheca fuliginea*^49^ and inhibit sclerotia differentiation and growth in *Sclerotium rolfsii*^50^. Therefore, SA can have an indirect impact on fungal growth and development.

Our obtained data on the biological aspects, including the daily survival percentage of adult females of *A. gossypii* Glover and their first day-born nymphs, were potently affected by the LC_20_ of HAL extract more than buprofezin and the control treatment. Previous studies confirmed the current study on the detected moieties of HAL extract represented in gallic acid, quercetin, apigenin, ellagic acid, and salicylic acid as a primary cause of the lethal activity against *A. gossypii* Glover^51–55^. Ellagic acid was found to be the augmented cause of the lethal effect on the larval and pupal stages of *Spodoptera litura* Fabricius^53^. In addition, apigenin was detected as the source of the toxic effect on the larval stage of *Culex quinquefasciatus* due to its capability to inhibit AChE1 and damage the epithelial cells in the mid-gut of the developmental stages^55^. Moreover, quercetin-3-O-glucoside showed vigorous toxic effects against *Aphis craccivora*^54^. Certain concentrations of salicylic acid also could manifest slight reductions in the populations of *A. gossypii* Glover and *Bemisia tabaci* Gennadius, mainly attributed to the leverage of the phenolic compounds in the treated leaf contents^52^. On the other hand, The GC–MS analysis of HAL extract detected specific unsaturated carboxylic fatty acids represented in palmitic acid, n-hexadecanoic acid, 9,12-octadecadienoic acid (*Z,Z*)-, 11-cis-Octadecenoic acid, known as cis-vaccenic acid, and trans-13-octadecenoic acid that may be causative factors of the insecticidal activity against *A. gossypii* Glover. This assumption meets previous studies on the insecticidal activity of identical fatty acids on various insect pests^56–58^. It is well known that there is a correlation between the multiplicity of the double bonds in these fatty acid chains and the larvacidal leverages against *S. littoralis* Boisd^59,60^. Furthermore, the free carboxyl group in fatty acids could deactivate the systemic stability of cells^61^ and consequently cause the neat carboxylic fatty acids to attain more toxic effects than their counterparts in the methylated form^59^.

In our study, HAL extract was comparable to buprofezin at their sub-lethal concentrations (LC_20_) in prolonging the longevity of the 24h-born nymphs from treated adult females and the total life cycle compared to the control. The presence of the detected moieties of ellagic acid, rutin, and chrysin in HAL extract may be in line with investigations in previous studies^53,62,63^. Where ellagic acid adversely prolonged the intervals of the developmental stages of *S. litura* Fabricius^53^. In addition, the flavonoid rutin was characterized by an obvious prolongation in the developmental duration of larvae and the total life cycle of *Spodoptera frugiperda* J.E. Smith (Lepidoptera: Noctuidae)^62^. Chrysin is also thought to possess a prolonged effect on the larval stage and total developmental period of *Zeugodacus cucurbitae* Coquillett (Diptera: Tephritidae)^63^.

Regarding the data on the biological aspects of adult females of *A. gossypii* Glover, potent reductions for *R. indica* extract at LC_20_ were exhibited in the daily nymph production per female during the 72 h of exposure. The extract of HAL also had a significant reduction in the total reproductive rate, more than buprofezin, but was comparable to buprofezin on the daily reproductive rate and total reproductive period compared to the control. These results might be correlated to the content of chrysin in the extract of *R. indica,* as it was previously explored for its role in inhibiting the percentage of adult emergence of *Z. cucurbitae* Coquillett^63^. Additionally, flavonoids such as acacetin-7-O-glycoside that were found in extracts of *Robinia pseudoacacia* L. Fabaceae might have biological effects on *Myzus persicae* Sulzer (Homoptera: Aphididae)^64^.

The current study on the olfactory response of adult aphids showed that *R. indica* extract possessed the lowest attraction percentage on aphids and increased in buprofezin and more in the control at the 4th h of exposure. These findings were tuned to the presence of some phytochemical components in *R. indica* extract, such as gallic acid and epicatechin^65,66^. Gallic acid was found to induce epigallocatechin-3-gallate to promote direct defense in the treated plant by stimulating the signals of jasmonic acid^66^. Flavonoid (+)-catechin also exhibited potent repellency and antifeedant activity against rubber termites, *Coptotermes curvignathus* Holmgren (Isoptera: Rhinotermitidae)^65^.

In general, the results of our study indicate that the HAL extract under examination exhibits promising properties as an organic fungicide for combating *R. solani* and *F. verticillioides*. Additionally, it shows potential as an insecticide for eradicating cotton aphids. Nevertheless, additional research is necessary to determine the precise mode of action and probable applications of the extract in the agro-fields, thereby warranting further investigation.

## Conclusions

To sum up, the current investigation assessed the insecticidal and antifungal characteristics of an acetone extract derived from the leaves of Indian hawthorn (HAL). The extract exhibited some efficacy in impeding the fungal development of *Rhizoctonia solani* but demonstrated greater effectiveness against *Fusarium verticillioides* in laboratory settings. HAL extract attained a rational toxic effect on *Aphis gossypii* Glover. The LC_20_ of HAL extract vigorously affected the nymph production of adult females of *Aphis gossypii* Glover over 72 h and their overall reproductive rate. This extract decreased the daily reproductive rate, reproductive period, and mean survival percentage. Nevertheless, the newly born nymphs from treated females with HAL extract faced a decline in the survival percentage and prolongations in the longevity of the nymphs and the overall life cycle of *A. gossypii* Glover. The olfactory choice of cotton aphids showed a low attraction response to HAL extract. Thence, HAL extract may be sensibly included in control programs of the cotton aphid. HPLC and GCMS analyses revealed that the HAL extract was rich in salicylic acid, ferulic acid, epicatechin, and *n*-hexadecanoic acid. These findings suggest that the HAL extract may serve as a natural and sustainable solution to manage fungal infestations in plants and combat *A. gossypii* Glover, offering a preferable alternative to insecticides that can adversely affect human health and the environment in the long run.

## Data Availability

The datasets used and/or analyzed during the current study are available from the corresponding author upon reasonable request.
